# Supplementary material for: Observation of geometric phase effect through backward angular oscillations in the H + HD → H2 + D reaction
Source: Nat Commun. 2024 Feb 24;15:1698. doi: 10.1038/s41467-024-45843-6 (PMC11258225; doi:10.1038/s41467-024-45843-6)
Supplement: Supplementary file 1 — Supplementary information [file 41467_2024_45843_MOESM1_ESM.pdf]

Supplementary information

Observation of geometric phase effect through backward angular oscillations in the $H + HD \rightarrow H_2 + D$ reaction

Shihao Li[#1], Jiayu Huang[#2,3], Zhibing Lu[1], Yiyang Shu[1], Wentao Chen[1], Daofu Yuan[1],

Tao Wang[4], Bina Fu[2,5], Zhaojun Zhang*[2,5,6], Xingan Wang*[1,5],

Dong H. Zhang*[2,4,5] and Xueming Yang*[2,4,5]

1. Department of Chemical Physics, University of Science and Technology of China, Hefei, 230026, China.

2. State Key Laboratory of Molecular Reaction Dynamics, Dalian Institute of Chemical Physics, Chinese Academy of Sciences, Dalian, 116023, China.

3. Department of Physics, Dalian University of Technology, Dalian 116024, China.

4. Department of Chemistry, College of Science, Southern University of Science and Technology, Shenzhen, 518055, China.

5. Hefei National Laboratory, Hefei, 230088, China.

6. University of Chinese Academy of Sciences, Beijing, 100049, China.

# These authors contributed equally in this work

Corresponding authors:

zhangzhj@dicp.ac.cn, xawang@ustc.edu.cn, zhangdh@dicp.ac.cn, xmyang@dicp.ac.cn

This PDF file includes

Supplementary sections 1- 4

Supplementary Figs.1- 5

Supplementary Note 1 | Experiment method

Herein, we present the experimental setup and new improvements in the crossed molecular beams study of the H + HD → H_2 + D reaction at a collision energy of 1.72 eV. The H-atom beam was generated by the photolysis of HBr molecules using a 213 nm laser light (fifth harmonic generation of a Nd: YAG laser, Continuum, Powerlite 9020). The intensity of the photolysis laser light is 25 - 30 mJ/pulse. The HBr molecular beam was generated by the supersonic expansion from a pulsed valve (General Valve, Parker, serial 9). The stagnation pressure of HBr was 1 bar. By setting the polarization of 213 nm light to be vertically polarized, the faster H atom beam with a velocity of 19.80 km/s was selected, and it corresponds to the H + Br ($^2P_{3/2}$) dissociation channel. The HD molecules were cooled to the liquid nitrogen temperature and supersonically expanded through the other pulsed valve (the Even-Lavie valve, Lamid). The velocity of the HD molecule beam was 1.24 km/s, and about 97% of the HD molecules in the beam were populated in the ground rovibrational state HD (v = 0, j = 0)[1]. The two beam sources and the scattering region were differentially pumped to achieve a high vacuum environment, and the crossing angle was 160°. The product D atom was ionized by the 1+1' (121.6 nm + 364.5nm) near threshold resonance-enhanced multiphoton ionization method. The ionized D atom fragments were guided and accelerated by the ion optics. The ions flew through a time-of-flight tube and then were projected onto a position-sensitive, 70 mm diameter dual microchannel plate (MCP) coupled to a phosphor screen. A fast-pulsed voltage was applied on the MCP to perform the time sliced measurement. The position sensitive ion signals were converted to visible lights by the phosphor screen (P43) attached to the MCP. The images were captured and recorded by a charge-coupled device (CCD) camera (LaVision pro plus 2M). A real time ion event counting method was applied during the data acquisition of ion images. In order to achieve the equal detection efficiency of D atom products with different velocity, the wavelength of the probe laser was scanned back and forth to tune the wavelength of the VUV laser beam to cover the whole Doppler profile of the D atom products during the measurement.

In particular, in the experiment, we employ a new ion optics with an optimized base that can ensure that the electrodes of the ion lens are precisely parallel to the MCP detector. Meanwhile, the liquid nitrogen cooling was introduced through a new cooper shielding cylinder to cool the temperature of the detection region, thereby improving the vacuum of the reaction center and the

signal-to-noise ratio. With the current improved velocity map ion imaging (VMI) method, we have acquired the rotational-state resolved product H_2 in the backward scattering direction.

Supplementary Note 2 | Data Analysis

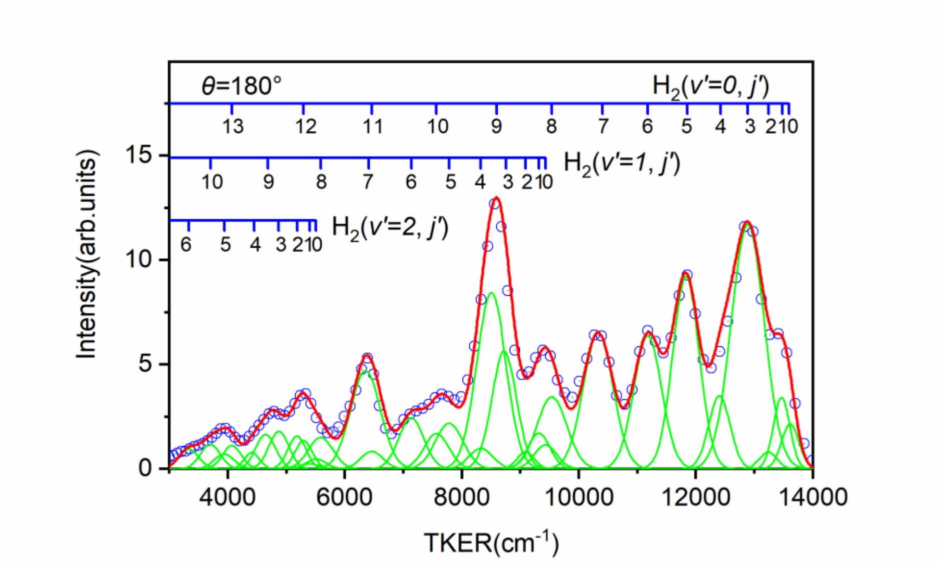

Supplementary Fig.1. The backward (180°) scattering total kinetic energy releases of H + HD reaction at the collision energy of 1.72 eV. The experimental data is presented as blue circles. The green and red lines denote the fitting for each rotational state (green line) and overall spectrum (red line) respectively. Energy combs (blue bars) are used to label the position of the rovibrational states of the H_2 products. Source data are provided as a Source Datafile.

In the backward scattering direction (180°) of the velocity image, a velocity distribution can be taken from zero to the maximum velocity of the product D atom. With the laws of conservation of energy and momentum, the velocity distribution of the D atom can be converted into the total kinetic energy release (TKER). A rotational state-resolved TKER spectrum in the backward scattering direction is obtained. By fitting the TKER spectra in each scattering angle, the ro-vibrational resolved differential cross sections for co-product H_2 are derived over the full scattering angles. Fast forward oscillations were clearly observed for H_2 (v'=0, j'=3, 5) states in Supplementary Fig.2. It enables the high accuracy and reliability when extracting the state resolved DCS by fitting the TKER spectra. For the DCSs shown in Fig. 2, the measurement error (due to counting statistics) in the current experiment is about 3% (Standard Deviation divided by the average value, SD/AVG)).

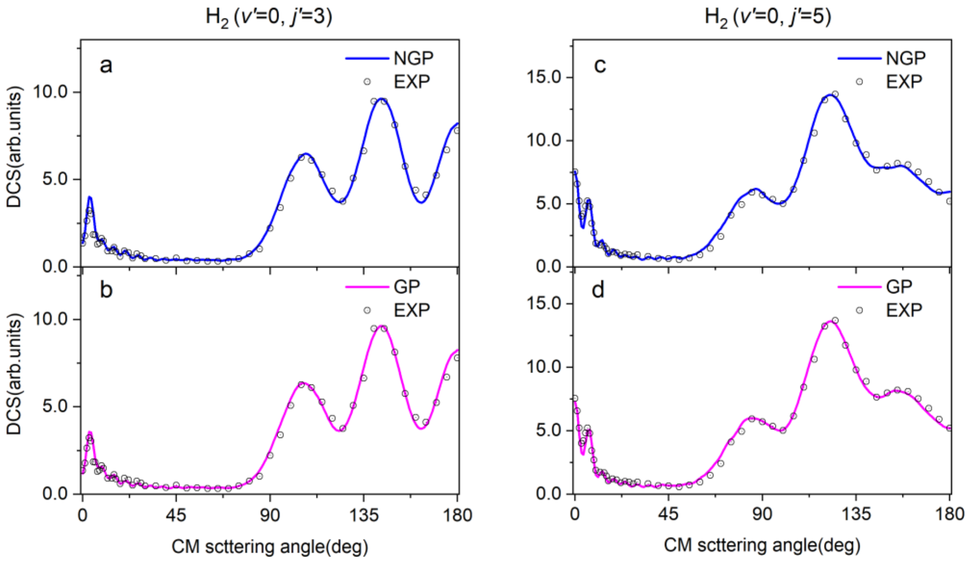

Supplementary Fig.2. Experimental (EXP) and theoretical (NGP and GP) angular distributions of the H_2 (*v'* = 0, *j'* = 3, 5) products. (a) and (b) show the results for *v'*=0, *j'*=3 state; (c) and (d) show the results for *v'*=0, *j'*=5 state of H_2 product. Source data are provided as a Source Data file.

Supplementary Note 3 | Quantum scattering calculations

The state-to-state reaction probabilities were extracted by the product-coordinate-based wave packet method. We prepared an initial wave function in reactant coordinates. A coordinate transformation was then carried out to transfer the wave packet from the reactant coordinates to the D + H_2 product coordinates. After the transformation, we propagated the wave packet for additional 900 iterations with Δt = 10 in the product coordinates. The application of the vector potential in the present work is based on a newly developed method which we referred to as the diabatic version of vector potential (DV-VP) approach. The new method merely takes the same computational cost as a diabatic calculation, significantly reducing the computational efforts required for the inclusion of the GP. For a detailed description of the DV-VP method, please refer to the prior work[2].

We applied a total of 161 sine basis functions (including 80 for the interaction region) in the R range of 0.1–14.0 bohr, a total of 110 potential optimized discrete variable representation basis functions (including 10 for the asymptotic region) in the r range of 0.4–11.8 bohr, and 110 rotational basis functions to converge calculation results. The range of the total angular momentum was taken as $0 \leq J \leq 45$, and all helicity channels were included. Damping functions were employed to prevent the wave packet from reflecting back from the boundaries in R_a = 10.5 bohr and r_a= 9.8 bohr.

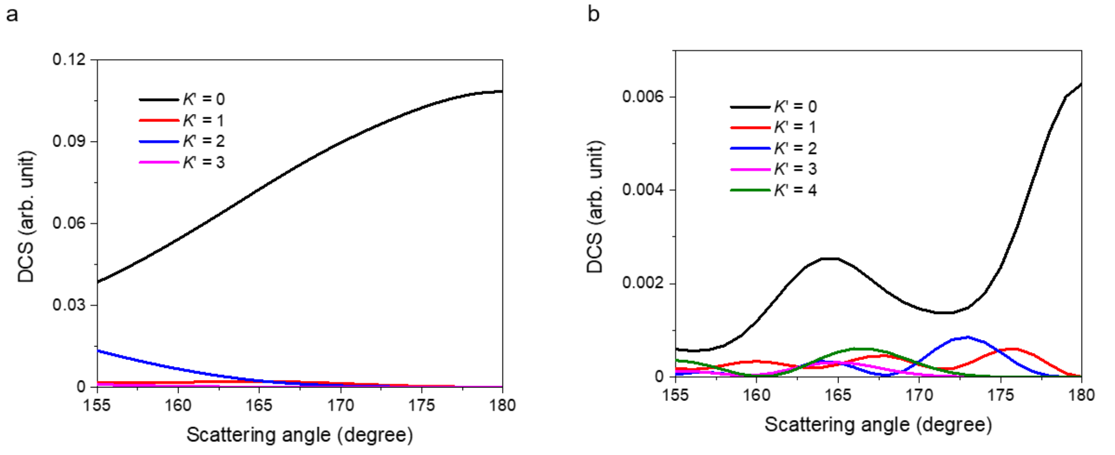

Supplementary Fig. 3. Theoretical analysis for product H_2 ($v' = 2, j' = 3$) generated through Path 1 and Path 2 in the backward scattering direction. (a) and (b) are K' quantum-number-specific DCSs for Path 1 and Path 2 product. Source data are provided as a Source Data file.

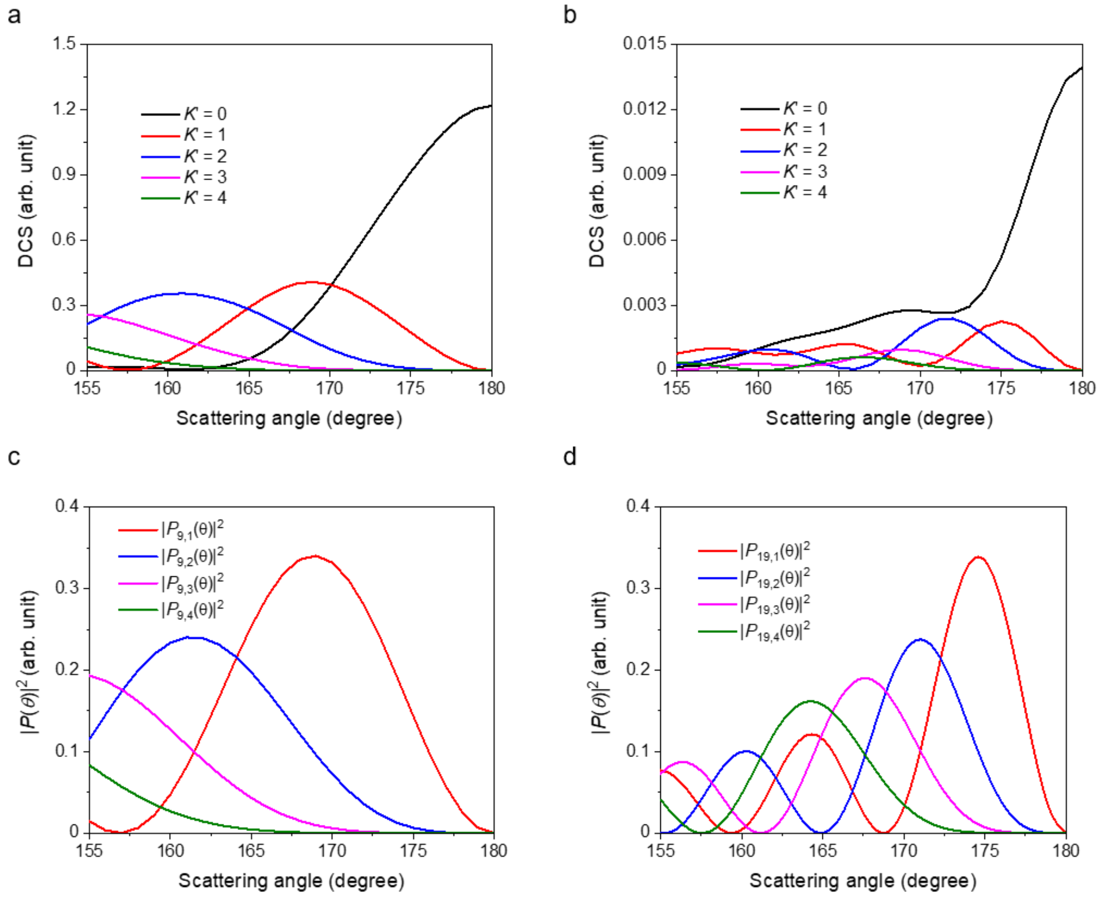

Supplementary Fig. 4. Theoretical analysis for product H_2 ($v' = 0, j' = 9$) generated through Path 1 and Path 2 in the backward scattering direction. (a) and (b) are K' quantum-number-specific DCSs for Path 1 and Path 2 products. (c) Square moduli of the associated Legendre polynomials $|P_{J,\,K'}(\theta)|^2$ with $J = 9$ and $K = 1, 2, 3, 4$. (d) Square moduli of $|P_{J,\,K'}(\theta)|^2$ with $J = 19$ and $K = 1, 2, 3$. Source data are provided as a Source Data file.

Supplementary Note 4 | Classical trajectories calculations

To confirm the reaction mechanism in the classical dynamics picture, quasi-classical trajectory (QCT) calculations were performed on the adiabatic BKMP2 PES. Although these simulations are unable to predict quantum effects such as the GP, they are expected to accurately capture the underlying dynamics, which are essentially semiclassical. The snapshots of a representative QCT trajectory of the path 2 mechanism for backward scattered H_2 ($v' = 2, j' = 3$) product are shown in Supplementary Fig. 5 in the sequence of (a)-(e). In the trajectory, the incoming H′ atom approaches HD molecule and passes the first H′-D-H linear transition state. As the HD molecular stretches, H′ atom inserts between H-D bond by experiencing the second D-H′-H transition state. Supplementary Fig. 5(f) shows the same trajectory in hyperspherical coordinate, clearly illustrating the roaming insertion nature of path 2.

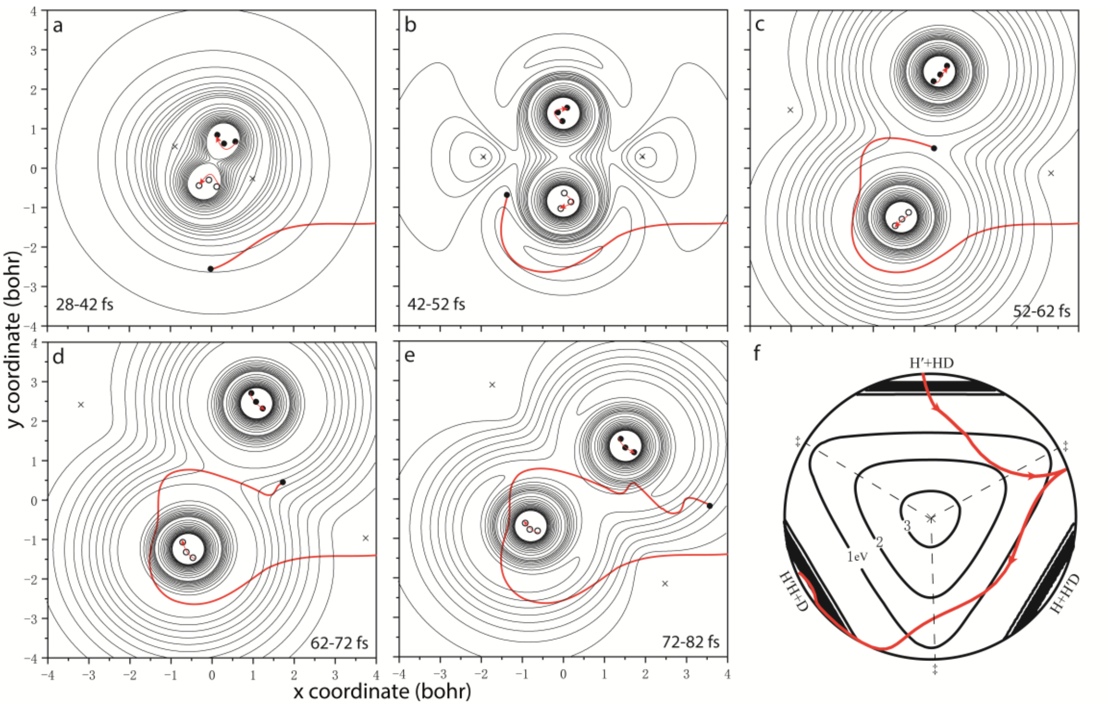

Supplementary Fig. 5. Representative classical trajectories for the H + HD (v = 0, j = 0) → H_2 (v' = 2, j' = 3) + D reaction in the backward scattering direction (161°) for reaction path 2 at the collision energy 1.72 eV. In a-e, the black dot denotes the H atom and the black circle denotes the D atom. The red curves represent the trajectories of the incoming H atom. The locations of conical intersections are denoted by crosses (×). The potential energy surface contours are plotted at instant times 36.0, 46.0, 59.0, 65.0 and 75.0 fs for a-e. In panel f, the trajectory (in a-e) is plotted in hyperspherical coordinates, which passes through two transition states (‡). The corresponding dotted lines separate three different atom-diatom channels.

References

1. Yuan, D. *et al.* Direct observation of forward-scattering oscillations in the H + HD → H_2 + D reaction. *Nat. Chem.* **10**, 653-658 (2018).

2. Huang, J. & Zhang, D. H. An efficient way to incorporate the geometric phase in the time-dependent wave packet calculations in a diabatic representation. *J. Chem. Phys.* **153**, 141102 (2020)
